# Dr. John Daniel Mitchell

**Published:** 1915-09

**Authors:** 


**Dr. John Daniel Mitchell,** whose death June 27, at the age of 62 was announced in our last issue, is credited by the J. A. M. A. to New York University, 1876. He was a founder and for six years superintendent of the Steuben Sanitarium.

